# Sage in Hyperidrosis

**Published:** 1897-06

**Authors:** 


### SAGE IN HYPERIDROSIS.

Infusion of sage is again recommended for the treatment of hyperidrosis in tuberculous subjects as well as those suffering from leukemia, rheumatic polyarthritis and typhoid fever; in thirty-eight cases where it was tried there were only two failures. Steap forty-five grains of sage leaves in half a pint of water, and let the patient take a cupful in the morning, one during the course of the day, and still another before retiring at night—or the tincture of the leaves may be given in twenty-drop doses in the morning, and from twenty to forty drops at night. *Salvia officinalis* has a proper place in the front ranks of anti-sudorific remedies.—*Med. Week.*

